# The perception of healthcare quality of elderly in the city of Bari, South Italy

**DOI:** 10.1186/1472-6963-7-174

**Published:** 2007-10-24

**Authors:** Rosa Prato, Domenico Martinelli, Annarita Fusco, Annarita Panebianco, Pietro Luigi Lopalco, Cinzia Annatea Germinario, Michele Quarto

**Affiliations:** 1 Section of Hygiene Department of Scienze Mediche e del Lavoro, University of Foggia, Foggia, Italy; 2 Section of Hygiene Department of Scienze Biomediche e Oncologia Umana, University of Bari, Bari, Italy

## Abstract

**Background:**

In recent decades in Italy, as in all the industrialized nations, the proportion of elderly subjects in the total population is constantly on the increase. However the increased life expectancy is not always paralleled by a true improvement in the quality of life.

In this context, it is essential to analyze elderly real health needs and the responses to these needs, especially in terms of healthcare, that the territorial services are perceived to offer.

**Methods:**

In the period from June to September 2006 we selected randomly one General Practitioner (GP) for each district of the Bari Municipal Area and, form each GP, we randomly chose 25 patients over 65 years old (YO). We conducted phone interviews using a standard data collection questionnaire and, for each of the recruited subjects, the GP filled a data collection sheet.

**Results:**

Although the mean age (73.6 years) of the population under study was quite high, the general state of health was judged good both by the G P- and by their elderly patients (>75%).

Notably, the great majority of elderly patients considered the healthcare they receive to be satisfactory (>60%): in particular, the GP was the true point of reference for this slice of the population for strictly medical problems as well as for advice. On the contrary, the patients attributed little value to social services, which were poorly known and scarcely used (8.5%). Public hospital facilities played a central role in second level healthcare in more than 30% of cases; private facilities covered by public health insurance were also very important. As possible solutions to the problem of loneliness, 36.6% of the patients declared that they approved of nursing homes.

**Conclusion:**

Decision makers need to create services supporting the key role played by General Practitioners, who are well aware that their assistance is not sufficient to satisfy the health needs of the elderly.

## Background

In recent decades in Italy, as in all the industrialized nations, the proportion of elderly subjects in the total population is constantly increasing. The progressive aging of the population is the result of profound demographic changes that include both a falling birth rate and a consistent decline in the mortality rates for all causes [1-4].

Life expectancy at 60 years of age has increased by 2 years in the last 2 decades, and the number of people over 75 years of age has thus risen in the same period by 33.7%, nearly twenty fold higher than the overall increase in the Italian population (1.8%). Italy is the first nation in the world where the percentage of elderly subjects has exceeded that of young people less than 15 years of age (17.3% vs 14.5%). In the future this difference will surely widen. It is estimated that in the next ten years the number of young people between 15 and 34 years of age will decline by about 5 million due to declining fertility rates, while the number of elderly people will rise by 1.5 million. This progressive population aging is expected to reach a peak in 2030, the year when there are expected to be 15 million people over 75 years of age in Italy, accounting for 28% of the population [5]. This increased life expectancy is not paralleled by improvements in the quality of life, because advancing age often goes hand in hand with a loss of autonomy, aggravated by the presence of multiple disease and a condition of social isolation [6-13].

There is a widely felt need to explore the "world of senior citizens", to define the socio-demographic characteristics of this large slice of the population, identify their needs and provide decision makers with useful, efficacious support for political actions [14-16].

In this context, therefore, besides an objective and subjective investigation of the health of the elderly, it is essential to analyze their real health needs and the response to these needs, especially in terms of healthcare, that the territorial services are perceived to offer [17]. In the present study, this assessment has been made by comparing the subjective experience of patients over 65 years of age with that of their General Practioner (GP), who takes the main role in primary health care of elderly, in Italy [18-21].

## Methods

In the period June – September 2006 we performed a cluster sampling study among the GPs and their populations of registered patients over 65 years of age resident in the Bari Municipality Area. One GP for each municipal district (N = 15) was selected. 25 subjects aged over 65 years were selected from 11 of the 15 GP, 16 subjects from 1 GP, 15 subjects from 2 GPs, 10 subjects from 1 GP, making up a total sample of 331 subjects. In Italy every GP cannot take care of more than 1,500 patients aged more 14 years (people ≤ 14 years are followed by family paediatricians); therefore they can have a number of patients less than 1,500. For those GPs with less than 1,500 registered patients, the number of patients represented about 2.5% of the total number of patients over 65). All the GPs and subjects were selected randomly.

The investigation was conducted by phone interview using a standard data collection questionnaire administered to each study subject, after obtaining informed consent. For each of the recruited subjects, the GP filled a data collection sheet. In the patients study sample, the questions regarded the perception of their psychophysical state of health, present disabilities, drugs prescribed, health assistance received [22-25]. The first part of the questionnaire indicated the name of the person who answered the telephone (study subject or, in cases of severe physical and/or mental disability, another subject) and the personal details of the enrolled subject.

The following sections were included in the questionnaire:

- state of health;

- loneliness;

- drugs taken and administration route;

- disabilities and autonomy in carrying out daily activities;

- degree of satisfaction with healthcare received;

- knowledge of available health services (health benefit contribution to supply drugs, integrated home assistance, emergency phone service, etc);

- frequency and type of consultation of the GP;

- method, frequency and facility consulted for analyses and other diagnostic tests;

- method, frequency and facility consulted for specialist visits and physiotherapy.

In the same context, possible areas of intervention and initiatives serving to better respond to the identified needs were investigated: opinions on facilities like nursing homes, knowledge of the concept of "group apartment", frequency of recreation activities, proposals for improving the situation.

At the same time, the GPs' opinions of the same topics were assessed (every GP were answering questions about each of his own patients). The questionnaire included 10 topics:

- state of health of the patient;

- main disabling disease/s;

- frequency and reason for ambulatory visits;

- degree of satisfaction with the healthcare received by the patients;

- facility consulted for laboratory tests, instrumental investigations and specialist visits, and reason for their choice;

- need and indication for other types of assistance, apart from those supplied.

GPs were not allowed to access to their patient interviews. In the same way, the patients did not know about GPs opinions.

The data collected were stored in a database built with File Maker Pro 7.0 software for MacOs X and processed with Epi Info 3.3. Prevalence rates and 95% confidence intervals (95% CI) were calculated taking into account the *design effect *due to the cluster sampling [26]. Epi Info 6.0.4 software was used for calculation. Contingency tables were plotted for comparisons between categorical variables, calculating the Chi square (χ^2^) value; values of p <0.05 were considered significant.

## Results

### Study population

The National Statistics Institute current data for 1 January 2005 were used to estimate the sample size of elderly (over 65) resident in the Bari municipality [27]. The total population of over 65 year olds resident in the Bari municipality equals 59,500 people (18.1% of the general population).

The study sample consisted of 15 GPs and 331 elderly patients. GPs were not able to answer about 16 patients, therefore the final number of questionnaires completed by GPs was 315. Patients' willingness to participate in the study was high: only 4.8% refused consent, while a person other than the subject answered for 34.1% of the subjects enrolled.

Patients mean age was 76.3 years (median = 75 years; mode = 72, SD = ± 6.08; range: 66–98); 53.8% were male and 46.2% female (Table 1); 62.5% were married, 28.7% widowed, 6% unmarried, 2.7% divorced; 51% lived with their spouse, 20.3% with a son/daughter, 18% alone, 3.4% with a carer (7.3% with others); 38.7% had attended primary school, 19.9% middle school, 16.9% high school, 12.4% a university, while 12.1% had no scholastic attendance certificate. As to working activities, about one third of the sample were housewives (29.6%), 70.5% declared that they received a retirement pension, 24.5% social benefit and 5% a carer contribution.

**Table 1:** Sample distribution, by sex and age

	Female		Male		Total	
	
Age	N	%	N	%	N	%
65 – 74	71	46.4	79	44.38	150	45.32
75 – 84	66	43.1	76	42.70	142	42.90
≥ 85	16	10.5	23	12.92	39	11.78

Total	153	-	178	-	331	-

### State of health

Among the patients, 27.8% defined their state of health as good or very good, 49.2% *(95% CI: 45.6–52.9) *as moderate and 21.5% *(95% CI: 16.9–26.7) *as poor; 1.2% *(95% CI: 0.2–4.9) *answered "I don't know". For their GPs 2.9% *(95% CI: 0–5.9) *of the selected sample was in very good, 26% *(95% CI: 16.6–35.4) *in good, 51.4% *(95% CI: 43.7–59) *in moderate and 18.4% in poor health *(95% CI: 11.9–24.9)*; 1.3% *(95% CI: 0.2–4.9) *answered "I don't know". The difference between the perception of health of the patients and their GPs was not significant (p >0.05 – Table 2). The most frequently diagnosed disease was hypertension (57%; *95% CI: 52.6–62.1*), followed by arthritis (38.7%; *95% CI: 30.4–47.6*), cardiovascular disease (32.6%; *95% CI: 26.4–39.5*), diabetes (19.3%; *95% CI: 14.2–25.7*), respiratory diseases (15.7%, *95% CI: 11.3–21.3*%).

**Table 2:** State of health, by patients and GPs opinion

	Patients		GPs	
	
State of health	n	% *(95% CI)*	n	% *(95% CI)*
Very good	21	6.3 *(3.7–10.6)*	9	2,9 *(0–5.9)*
Good	73	21.7 *(18–26)*	82	26 *(16.6–35.4)*
Moderate	166	49.2 *(45.6–52.9)*	162	51.4 *(43.7–59)*
Poor	71	21.4 *(16.9–26.7)*	58	18.4 *(11.9–24.9)*
Don't know	4	1.2 *(0.2–4.9)*	4	1.3 *(0.2–4.9)*

Total	331	-	315	-

**Figure 1:**
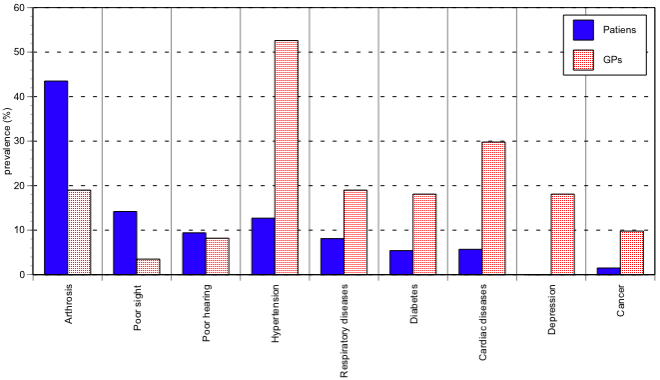
Distribution of diseases defined as limiting, by the patient and by the GP.

Limitations to carry on any activities (indoor and outdoor) due to a health problem were referred by 61.9% *(95% CI: 53.3–69.8) *of the patients. According to the patients, the main health problems causing these limitations are: arthrosis (in particular, problems in walking) for 43.5% *(95% CI: 37.4–49.8) *of the sample, poor sight for 14.2 *(95% CI: 8.6–22.4) *and hearing for 9.4% *(95% CI: 5–16.4)*, hypertension for 12.7% *(95% CI: 8.5–18.2)*. According to their GPs, the main causes of disability are hypertension in 52.6% *(95% CI: 44.9–60.2) *of cases, arthrosis (in particular, problems in walking) in 19% *(95% CI: 13.9–25.4)*, heart problems in 29.8% *(95% CI: 22–38.9)*, depression in 18.9% *(95% CI: 12.6–25.1)*, diabetes in 18.1% *(95% CI: 14.6–22.1)*. There is a significant difference between the GPs' and the patients' opinions (χ^2 ^= 188.27, p <0.001 – Figure 1).

### Loneliness

When asked about feeling lonely, 9.7% (95% CI: 5.6–15.9) of the patients interviewed reported that they felt lonely "always", 45.6% (95% CI: 34.7–56.9) "sometimes", while 35% (95% CI: 28.7–42) declared that they "never" felt lonely. 9.7% (95% CI: 5.5–16.1) answered "I don't know".

### Drugs

78.2% (95% CI: 71.9–83.5) of the sample is under pharmacological therapy following their doctor's instructions: daily by 78,2% of the patients, periodically by 4.8%, at need by 11.3%. The remaining 5.7% did not answer the question. Some changes in the prescribed regimen were introduced by 14.2% (95% CI: 10–19.6) of those interviewed, while 4.2% (95% CI: 2–8.2) did not follow the GPs' instructions because too "many drugs are considered harmful" (50%), "not all are equally useful" (12.5%), because the patient forgot to take them (33.3%) or other (4.2%). The drugs most frequently taken without a medical prescription were: NSAIDS (81.5%), gastroprotectors, antidiabetics and laxatives (all in 3,1% of cases), antihypertensives, vasoprotectors and antibiotics (all in 1.5% of cases), all types of drugs (4.7%). 3.4% (95% CI: 1.6–6.5) didn't answer the question.

### Disabilities

12.7% (95% CI: 8.4–18.6) of the patients see well, while, due to declining sensory powers, 72.8% (95% CI: 65.3–79.2) need glasses to see well, 11.8% (95% CI: 8.5–16.1) refer that they cannot see well and 2.7% (95% CI: 1.4–5) have serious sight impairment. 67.7% of the sample (95% CI: 59.5–74.9) hears well, while 25.4% (95% CI: 19.8–31.9) have difficulty in hearing and 6.9% (95% CI: 3–14.2) are frankly "deaf". In addition, 67% (95% CI: 58.1–74.9) of cases do not have difficulty in chewing harder foods, 24.6% (95% CI: 19.1–30.7) have difficulty, and 8.4% (95% CI: 4.1–16) are unable to do so.

As regards daily activities, 8.7% (95% CI: 6.5–11.7) of the sample need help to move from one room to another, 10.3% (95% CI: 8.1–12.9) to go to the bathroom, 11.9% (95% CI: 8.4–16.2) to wash, 18.7% (95% CI: 13.6–23) to take a bath or shower, 15.1% (95% CI: 11.3–19.8) to dress, 14.2% (95% CI: 11.3–17.6) to climb up/down the stairs. They receive help from a son/daughter in 39.6% and from their spouse in 30.8% of cases, 21,6% from others. Although only 21.8% (95% CI: 14–31.9) referred regular physical activity, 55.6% (95% CI: 46.3–64.5) of the sample goes out every day and 68.6% of the latter declared that they do not need to be accompanied.

### Assessment of healthcare

Among the patients, 17.5% (95% IC: 11–26.4) rated the healthcare they receive as very good, 43.1% (95% IC: 35.2–52.1) as good, 28.7% (95% IC: 22.4–35.9) as moderate, 7.8% (95% IC: 4.5–13.2) as poor; 3.6% (95% IC: 1.6–6.5) did not know how to answer the question. According to their GPs, 16.5% (95% IC: 6–35.8) of the patients are very satisfied with the healthcare received, 57.5% (95% IC: 46.1–67.5) are moderately satisfied, 2.2% (95% IC: 0.8–5.5) are dissatisfied; the GP cannot give an opinion about 23.2% (95% IC: 13.5–36.4) of cases. There is a significant difference between these answers (χ^2 ^= 76.1, p <0.001), partly due to the GPs' difficulty in judging their patients' opinions (Table 3).

**Table 3:** Assessment of healthcare, by patients and GPs' opinion

	Patients		GPs	
	
Assessment of healthcare	n	% *(95% CI)*	n	% *(95% CI)*
Very good	54	17.5 *(11–26.4)*	52	16,5 *(6–35.8)*
Good	144	43.5 *(35.2–52.1)*		
Moderate	95	28.6 *(22.4–35.9)*	181	57,1 *(46.1–67.5)*
Poor	26	7.8 *(4.5–13.2)*	7	2,2 *(0.8–5.5)*
Don't know	12	3.6 *(1.6–6.5)*	75	23,2 *(13.5–36.4)*

Total	331	-	315	

### Social Services

As regards patients' knowledge of health assistance, 8.9% (95% IC: 5.3–14.4) know about health benefits, 23.9% (95% IC: 19.7–28.6) about home care service integrated and 43.2% (95% IC: 35.4–51.3) about emergency telephone assistance. Recourse to the municipal social services was made by 7.6% (95% IC: 4.7–11.7) of the patients. Among them 42.9% were satisfied with the attention received, whereas 39.3% complained of long waiting lists and bureaucratic difficulties; the remaining 17.8% of the subjects interviewed did not know how to answer the question.

### General Practitioners

In the last year, 1.8% (95% CI: 0.3–7.2) of the interviewed patients needed to visit their GP every day – this figure was 0.6% (95% CI: 0.1–2.1) according to the GPs 14.5% (95% CI: 8.4–23.5) of cases once a week – 10.5% (95% CI: 5.3–19.1) according to the GPs, 54.1% (95% CI: 47.2–60.8) of cases once a month – 33% (95% CI: 24.9–42.2) according to the GPs, 17.5% (95% CI: 13–23.1) of cases at least once every 3 months – 24.1% (95% CI: 18.7–30.4) according to the GPs, 6.6% (95% CI: 4–10.7) of cases once every 6 months – 13.7% (95% CI: 7.8–22.6) according to the GPs, 4.6% (95% CI: 2.8–7.2) of cases never – 18.1% (95% CI: 12.7–24.2) according to the GPs; 0.9% (95% CI: 0.4–2) didn't answer the question. There is a significant difference between the answers given by the patients and the ones given by their GPs (χ^2 ^= 58.3, p < 0.001).

For the patients, the main reasons for going to their GP were the onset of new symptoms in 30.5% (95% CI: 23.2–38.8) of cases – 37.5% (95% CI: 25.3–51.4) according to the GPs; known diseases in 47.1% (95% CI: 40.7–53.6) of cases – 48.5% (95% CI: 35.6–61.7) according to the GPs; drug prescriptions in 83.1% (95% CI: 72.1–90.4) of cases – 47.6% (95% CI: 32.6–63) according to the GPs; to ask advice in 10.9% (95% CI: 6.3–17.9) of cases – 26.3% (95% CI: 15–41.5) according to the GPs. Again, there was a significant difference between the opinions of the two groups (χ^2 ^= 38.2, p < 0.001 – Table 4).

**Table 4:** Main reasons why patients need to visit their GP, by patients and GPs' opinion

	Patients	GPs
Main reasons	Prevalence % *(95% CI)*	Prevalence % *(95% CI)*

To ask advise	10.9 *(6.3–17.9)*	26.3 *(15–41.5)*
Known diseases	47.1 *(40.7–53.6)*	48.5 *(35.6–61.7)*
Drug description	83.1 *(72.1–90.4)*	47.6 *(32.6–63)*
New symptoms	30.5 *(23.2–38.8)*	37.5 *(25.3–51.4)*
Number of answers	331	315

Contacts with the GP were by telephone for 40% (95% CI: 30.7–49.8) of the sample, at the ambulatory office for 68.9% (95% CI: 62.8–74.4), by home visit for 16.9% (95% CI: 7.8–31.9), while a person other than the patient went to the doctor for 16.6% (95% CI: 12.1–22.3) of the sample. 51.9% (95% CI: 46.2–57.7) of the patients declared that they were satisfied with their GP's medical care, while reasons for dissatisfaction or dissuading the patient from applying to the GP were long waiting times (12.1%; 95% CI: 8.8–16.2), inconvenient opening hours (2.4%; 95% CI: 0.76–6.6), the distance from home (4.83%; 95% CI: 2.3–9.4).

### Laboratory tests, instrumental investigations, specialist visits, physiotherapy

**Figure 2:**
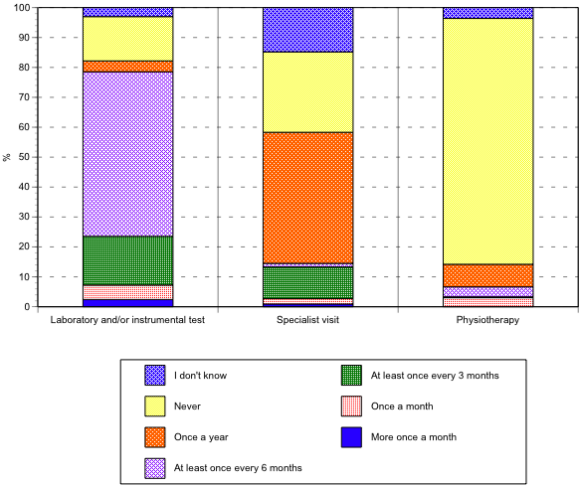
Percentage distribution of recourse to laboratory, diagnostic-instrumental tests, specialist visits, physiotherapy cycles.

In the previous year, 55% (95% CI: 47.1–62.6) of the patients had undergone a laboratory and/or instrumental test at least once every 6 months, and 43.8% (95% CI: 36.3–51.6) had had a specialist visit at least once. Again in the previous year, most of the patients (82,2%; 95% CI: 77.8–85.8) did not undergo or did not need physiotherapy, while 7.5% (95% CI: 5.6–10) had undergone at least one session (Figure 2).

Routine laboratory tests were done in a public facility (For laboratory tests hospital and public facility were considered all together) for nearly 42.5% (95% CI: 34.3–51.3) of the patients, mainly because they followed the GP's advice (29.8%) or had more confidence in it (31.2%); private facilities covered by public health insurance were used by 27.5% (95% CI: 21.3–34.7): because they were nearer home for 41.8% of the sample, or were indicated by the GP (28.6%); the 13.9% (95% CI: 9.9–9.1) of the sample consulted a private facility, largely because they thought waiting lists were shorter (34%), or because the facility was nearer home (34%).

Diagnostic-instrumental tests were performed at a hospital facility for 29.9% (95% CI: 26–34) of the patients because they had more confidence in it (32.3%), because it was advised by the GP (25.3%); at a private facility covered by public health insurance for 17.8% (95% CI: 12.6–24.5), largely because they thought waiting lists were shorter (27.3%), because it was advised by the GP (10.2%) or the facility was nearer home (12.6%); at a public "territorial" facility for 13.3% (95% CI: 8.7–19.6) because it was advised by the GP (43.2%) or was nearer home (22.7%). The 13.29% (95% CI: 8.6–19.7) of the sample went to a private facility because they had more confidence in it (38.6%) and thought the waiting lists were shorter (34.1%).

The specialist most frequently consulted was the cardiologist in 29.8% of cases, followed by the ophthalmologist in 16.3% of cases, the orthopedic surgeon in 11% and the urologist in 8.2% of cases; other specialists (the oncologist, endocrinologist, physiatrist, etc.) were consulted in 34.7% of cases.

For the specialist visit, 29% (95% CI: 24.9–33.4) of the sample went to a hospital facility, mainly because they had more confidence in it (43.8%) or it was advised by the GP (28.1%). Instead, 20.5% (95% CI: 16.3–25.4) went to a private facility because it was advised by the GP (36.8%) or they had more confidence in it (33.8%). A public territorial facility was chosen by 13.9% (95% CI: 10.1–18.7) of the sample because it was advised by the GP (41.3%) or nearer home (21.7%). The 7.8% of the sample (95% CI 5.3–11.3) consulted a specialist at a private facility covered by public health insurance because they found shorter waiting lists or it is near home (19.2%) or it was advised by the GP (26.9%).

**Figure 3:**
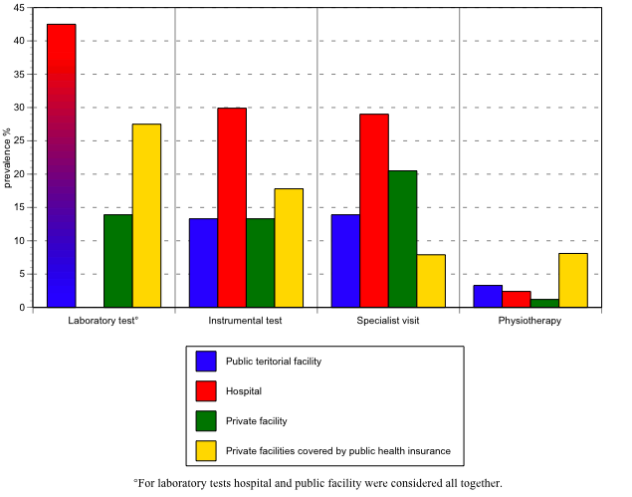
Percentage distribution of facilities consulted by the elderly patients interviewed, by type of procedure.

For a physiotherapy cycle 8.1% (95% CI: 5.9–11.1) of the sample chose a private facility covered by public health insurance because it was nearer home (38.5%) or advised by the GP (23.1%), while 1.2% (95% CI: 0.5–2.6) preferred a private facility because they had more confidence in it (100%) or because it was advised by the GP (75%); 3.3% (95% CI: 1.9–5.5) went to a territorial service because it was nearer home (36.4%) and 2.4% (95% CI: 1.3–4.2) went to hospital (Figure 3; Table 5).

**Table 5:** Reasons why the elderly patients chose the different facilities

		Public facility			Private facility		Private facility covered by public health insurance	
	
**Laboratory tests**^†^*		N (141)	*Prevalence %*		N (46)	*Prevalence %*	N (91)	*Prevalence %*
indicated by GP		42	*29,8%*		4	*8,7%*	26	*28,6%*
cost less		11	*7,8%*		-	*-*	3	*3,3%*
shorter waiting lists		12	*8,5%*		17	*37,0%*	25	*27,5%*
more confidence		44	*31,2%*		6	*13,0%*	14	*15,4%*
nearer home		30	*21,3%*		16	*34,8%*	38	*41,8%*
better treatment		7	*5,0%*		6	*13,0%*	6	*6,6%*
only one known		21	*14,9%*		1	*2,2%*	17	*18,7%*
								
	Public territorial facility		Hospital facility		Private facility		Private facility covered by public health insurance	
	
Instrumental investigations*	N (44)	*Prevalence %*	N (99)	*Prevalence %*	N (44)	*Prevalence %*	N (167)	*Prevalence %*

indicated by GP	19	*43,2%*	25	*25,3%*	5	*11,4%*	17	*10,2%*
cost less	5	*11,4%*	4	*4,0%*	1	*2,3%*	2	*1,2%*
shorter waiting lists	3	*6,8%*	20	*20,2%*	15	*34,1%*	21	*12,6%*
more confidence	8	*18,2%*	32	*32,3%*	17	*38,6%*	9	*5,4%*
nearer home	10	*22,7%*	14	*14,1%*	12	*27,3%*	16	*9,6%*
better treatment	2	*4,5%*	10	*10,1%*	5	*11,4%*	1	*0,6%*
only one known	3	*6,8%*	19	*19,2%*	2	*4,5%*	10	*6,0%*
								
Specialist visits*	N (46)	*Prevalence %*	N (96)	*Prevalence %*	N (68)	*Prevalence %*	N (26)	*Prevalence %*

indicated by GP	19	*41,3%*	27	*28,1%*	25	*36,8%*	7	*26,9%*
cost less	13	*28,3%*	4	*4,2%*	-	*-*	-	*-*
shorter waiting lists	1	*2,2%*	6	*6,3%*	12	*17,6%*	5	*19,2%*
more confidence	8	*17,4%*	42	*43,8%*	23	*33,8%*	3	*11,5%*
nearer home	10	*21,7%*	4	*4,2%*	2	*2,9%*	5	*19,2%*
better treatment	-	*-*	14	*14,6%*	9	*13,2%*	1	*3,8%*
only one known	4	*8,7%*	15	*15,6%*	8	*11,8%*	2	*7,7%*
								
Physiotherapy*	N (11)	*Prevalence %*	N (8)	*Prevalence %*	N (4)	*Prevalence %*	N (27)	*Prevalence %*

indicated by GP	3	*27,3%*	5	*62,5%*	3	*75,0%*	6	*23,1%*
cost less	-	*-*	4	*50,0%*	-	*-*	2	*7,7%*
shorter waiting lists	3	*27,3%*	-	*-*	-	*-*	-	*-*
more confidence	-	*-*	1	*12,5%*	4	*100,0%*	4	*15,4%*
nearer home	4	*36,4%*	-	*-*	1	*25,0%*	10	*38,5%*
better treatment	3	*27,3%*	-	*-*	1	*25,0%*	3	*11,5%*
only one known	3	*27,3%*	-	*-*	-	-	4	*15,4%*

^†^For laboratory tests, hospital and public facility were considered all together.

*Multiple-choice answers were possible.

### Proposals

According to the GPs enrolled in the study 28.9% (95% CI: 20.6–38.7) of the patients sampled needed further assistance, such as psychological support (25.3%), physiotherapy (25.3%), home care service integrated (16.4%), social assistance (15.1%), to go to a recreational centre (13.3%), or needed other support such as health benefits, or to live in a protected structure like a nursing home (4.6%).

As possible solutions to the problem of loneliness, 36.2% (95% CI: 27.8–45) of the patients declared that they approved of nursing homes, and 17.7% (95% CI: 11.9–20.3) had heard of the concept of a group apartment. Only 12.6% (95% CI: 9.3–17.1) of the sample went to any kind of meeting centre: of these 30.8% went to a municipal recreational centre, 24% to a religious association, 17.9% to a university for seniors, 5.1% did voluntary work, 22.2% were engaged in other activities (cultural, religious, recreational, etc.). 77% (95% CI: 69.6–83.2) were unaware of the possible existence of special services devoted to senior citizens in their residential quarter, while 60.7% (95% CI: 51.7–69.1) would like the municipality to organize activities for senior citizens in the municipal territory. Among the possible activities, those preferred were recreational centres and meeting places (52.4%) and services for the elderly (home care service integrated in 4.7%, healthcare in 5%, "old-sitters" like "help with shopping, buying medicines, etc." in 7%), 31.3 other.

## Discussion and Conclusion

The present study was conducted to assess the perception of healthcare of elderly patients over the age of 65 years resident in the city of Bari. It clearly depicts a fairly complex and multifaceted picture.

Although the mean age of the population under study was quite high, the general state of health was judged good both by the GPs and by the interviewed patients, as shown by the full agreement between the GPs' and their patients' opinions (79.3% versus 76%, aggregating all answers ranging from very good to moderate).

Instead, there was a difference between the diseases considered disabling by the patients and those referred as such by the GPs: the elderly feel "disabled" if they have difficulty in moving, or cannot see and/or hear well, while the GP considers disability to be related to difficulties in moving due to arthritis, but especially to systemic diseases such as hypertension and heart disease. The different perspective could be originated by the GPs knowledge of an objectively diagnosed health problem in the patient, while the patient himself perceives only the effects of this health problem, judging subjectively its effect on himself, such as the inability to carry any their normal activities out.

Notably, the great majority of elderly patients consider the healthcare they receive to be satisfactory (about 90% of cases aggregating answers ranging from very good to moderate). In particular, the GP is the true point of reference for this slice of the population for both strictly medical problems (83.1%for drug prescriptions, 47.1% for known diseases, 30.5% for new diseases), and for advice (10.9%, but 26.3% according to GPs opinions). The doctor's advice is then faithfully followed, as demonstrated also by the overall compliance with the prescribed therapy (78.2%). Instead, the patients attributed little value to social services, which were poorly known and above all little used (7.6%).

Public hospital facilities play a central role in second level healthcare in about 30% of cases, because they are considered more reliable. Private facilities covered by public health insurance are also considered important (used by about 15% of cases) and are preferred because they are usually nearer to home or have shorter waiting lists.

Although the importance of new contacts and mobility should be one of the targets in developing opportunities for elderly well-being [12], solutions to the issue of loneliness as a health problem are still largely lacking. Few senior citizens go to municipal facilities devoted to alleviating this problem(12.6%), either because probably such facilities are not widespread over the territory or because two out of three patients are unaware of their existence.

Satisfaction with care in older people is determined by several social, physical and financial factors probably interacting with each other. Especially the feeling loneliness, the degree of self-care capacity, poor overall health, anxiety, and sometimes poor financial resources are factors to be considered in the care of this specific population in order to preserve or improve their quality of life [13].

Decision makers therefore need to create services supporting the key role played by GPs, who, although conscious of the importance of the part they play in caring for the elderly, are also well aware that their assistance is not sufficient to satisfy their health needs. The real needs of this population could be guaranteed only by a continuous interchange and effective collaboration among the various healthcare facilities and social assistance-figures present throughout the territory.

## List of abbreviations

GPs: General Practitioners

## Competing interests

The author(s) declare that they have no competing interests.

## Authors' contributions

AF and AP carried out the survey and drafted the manuscript. RP and DM have made substantial contributions to conception and design of the study and performed the statistical analysis. CAG, PLL and MQ conceived the study as well as participating in its design and coordination.

All authors have read and approved the final manuscript.

## Pre-publication history

The pre-publication history for this paper can be accessed here:


